# Supplementary material for: Development and Evaluation of 2-Amino-7-Fluorophenazine 5,10-Dioxide Polymeric Micelles as Antitumoral Agents for 4T1 Breast Cancer
Source: Polymers (Basel). 2021 Dec 25;14(1):71. doi: 10.3390/polym14010071 (PMC8747360; doi:10.3390/polym14010071)
Supplement: Supplementary file 1 [file polymers-14-00071-s001.zip › polymers-1477558-supplementary.pdf]

SUPPLEMENTARY MATERIAL

Development and Evaluation of 2-Amino-7-Fluorophenazine 5,10-Dioxide Polymeric Micelles as Antitumoral Agents for 4T1 Breast Cancer

Nicole Lecot, Belén Dávila, Carina Sánchez, Marcelo Fernández, Mercedes González, Pablo Cabral, Hugo Cerecetto and Romina Glisoni

Figure S1. Linear average calibration curve of FNZ, in DMSO measuring at 296 nm. The curve was determined in triplicate (three independent samples, R^2= 0.9911). ……………… page 1

Figure S2. Plots of average size distributions obtained by DLS using Zetasizer family software v7.12. ……………… page 2

Figure S3. Plots of average size distributions obtained by TEM using FIJI platform analysis software. ……………… page 3

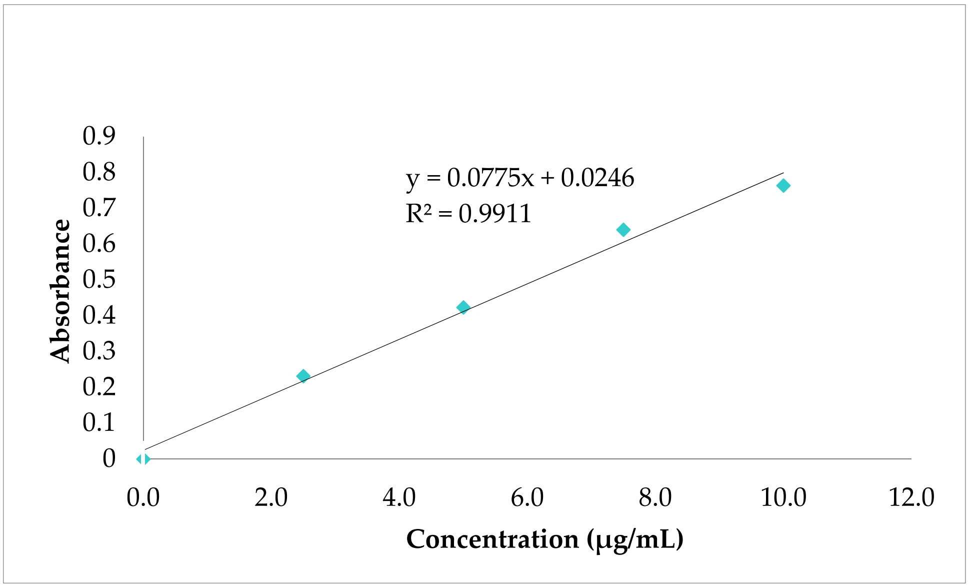

Figure S1. Linear average calibration curve of FNZ, in DMSO measuring at 296 nm. The curve was determined in triplicate (three independent samples, R^2= 0.9911).

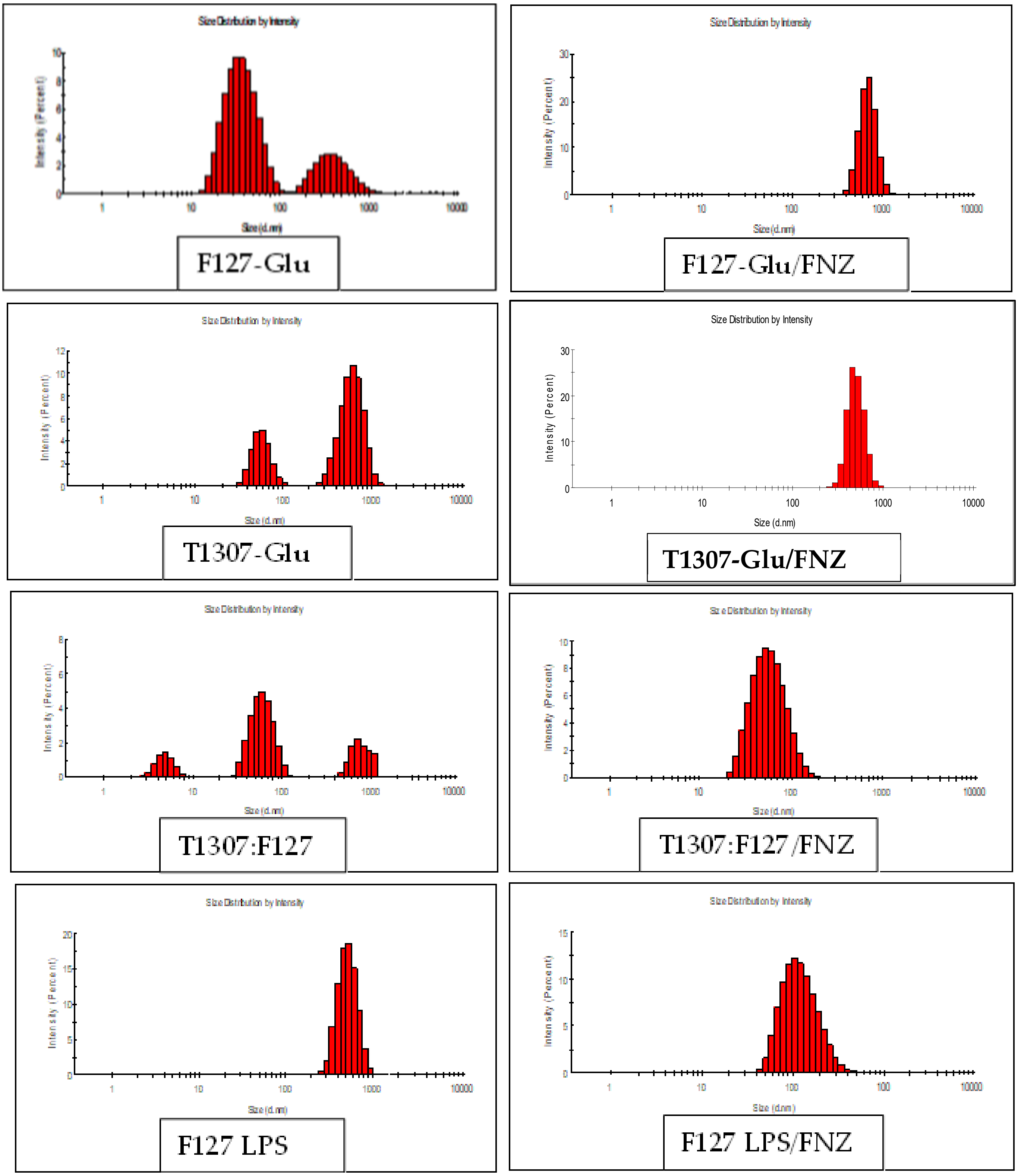

Figure S2. Plots of average size distributions obtained by DLS using Zetasizer family software v7.12.

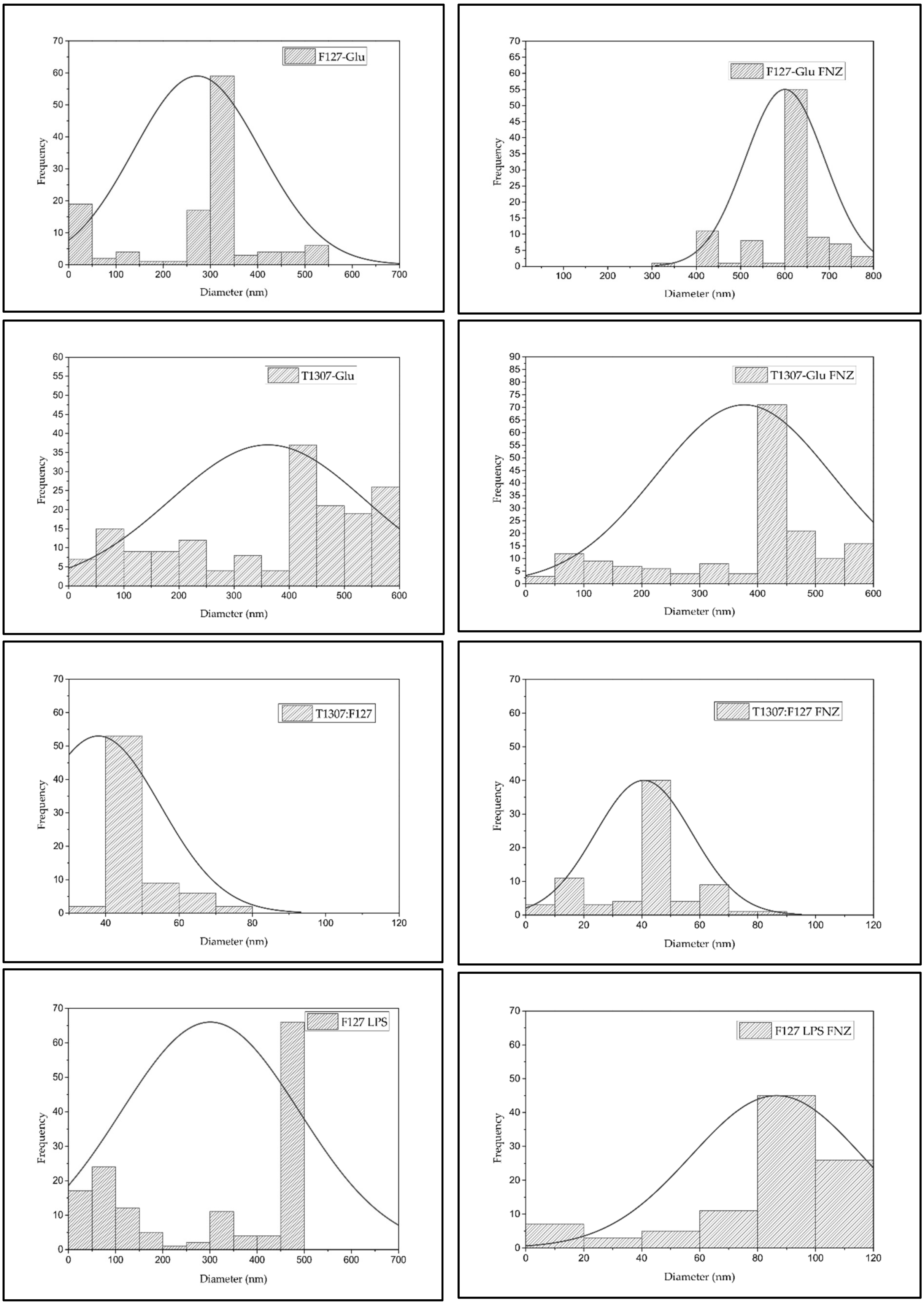

Figure S3. Plots of average size distributions obtained by TEM using FIJI platform analysis software.
